# Editorial: Women in surgical oncology vol II: 2022

**DOI:** 10.3389/fonc.2023.1223715

**Published:** 2023-06-08

**Authors:** Lidia Castagneto-Gissey

**Affiliations:** Department of Surgery, Sapienza University of Rome, Rome, Italy

**Keywords:** surgical oncology, women in surgery, breast cancer, ovarian cancer, sentinel lymph node

Female researchers in the medical field currently make up only a small minority, accounting for an estimated 29.3% of those who hold this job globally, with significant regional variations (1).

The UNESCO Institute for Statistics (UIS) is now working on new indicators to better understand the factors that influence women’s decisions to choose one field over another in response to the significant gender gap in scientific research. Women’s access to the scientific community may be restricted and discouraged for a number of reasons, including historical biases and gender preconceptions (2).

On the other hand, women currently represent over 50% of medical school graduates, and surgery has seen a steady rise in the number of female practitioners (3). The mentorship and education of women in this field are being improved by organizations and societies such as Women in Surgery across North America and Europe (3); yet, many obstacles still stand in the way of a successful career for women in surgery (4). Despite all the obstacles, women in surgery are academically and surgically on par with their male counterparts.

Following the success of the previous collection of research papers involving women in surgical oncology, the current Research Topic comprises further high-quality scientific contributions written by women who work in oncological surgery covering a variety of surgical oncology specialties, mainly concentrating on breast and ovarian cancer.

Among women of all ages, breast cancer is currently the most prevalent type of malignancy. Li et al. focused on identifying those populations of patients who could gain the greatest therapeutic benefits following adjuvant treatment, also evaluating prognostic factors among subjects with a T1-2N1miM0 breast cancer stage. They found that 5-year overall survival rates were significantly improved in those patients who underwent adjuvant radio-chemotherapy. Furthermore, patients with luminal A, luminal B, or basal-like subtypes, and T1c or T2 stage showed a greater response to adjuvant radiotherapy, while luminal A subtype, and T1b, T1c, or T2 stage had a higher beneficial response following adjuvant chemotherapy. On the other hand, Lan et al. aimed at determining the effect of neoadjuvant chemotherapy on the levels of reproductive hormones in order to assess the correlation of neoadjuvant chemotherapy with hormone receptor expression alterations and relapse-free survival in patients with breast cancer. Authors found that sex steroids (i.e. estradiol, progesterone, testosterone and dehydroepiandrosterone sulfate) significantly decreased after neoadjuvant chemotherapy in both pre and postmenopausal women. Additionally, there was a positive connection with progesterone receptor expression alteration and an inverse association between relapse-free survival and progesterone levels. Yang et al. retrospectively evaluated the prognostic value of fibrinogen-to-albumin ratio in 224 patients with triple-negative breast cancer. Fibrinogen-to-albumin ratio resulted to be a simple and inexpensive potential prognostic factor for disease-free survival and overall survival that could be implemented in clinical practice.

Zhao et al. describe an interesting case of breast cancer misdiagnosis. In fact, the patient had a history of lung cancer for which she received a percutaneous CT-guided needle biopsy several years earlier and this resulted in cancer cell seeding along the core needle tract, leading to a pulmonary metastasis at the level of the right breast, mimicking breast cancer.

Ovarian cancer is one amongst the most aggressive gynecologic cancers. Kampan et al. systematically reviewed the current literature regarding the prognostic value of sentinel lymph node mapping in patients with epithelial ovarian cancer. Authors found that the most common marker used is technetium-99m radiocolloid (Tc-99m) or indocyanine green, usually injected at the level of the ovarian ligaments. The overall detection rate of the sentinel lymph node, regardless of the method used, is as high as 84.5%. Authors propose a standardized procedure for ovarian cancer sentinel lymph node mapping, also encouraging further research which could lead to higher detection rates, characterization, and true positive rates, while for lower-volume metastases ultra-staging remains crucial.

Newton et al. carried out a systematic review of controlled clinical trials comparing patient-initiated follow up, particularly used during the COVID-19 pandemic, with another form of active follow up. They found no significant differences in overall survival rates, while one study found substantial differences in fear of cancer recurrence, which was greater in the patient-initiated follow up compared to the hospital-based follow up group. Hence, authors conclude that adequately powered randomized trials are necessary to determine the actual impact of patient-initiated follow up especially in the long term.

In order to change deeply rooted ideas, it is imperative to advance gender equality, crash stereotypes, and motivate women to work in STEM fields. Therefore, to highlight the achievements of female researchers in all fields of cancer, Frontiers in Surgical Oncology is glad to present this Research Topic. The studies presented here illustrate the breadth of oncology research in general and highlights recent advances in theory, experimentation, and methodology with applications to engaging current topics.

## Author contributions

The author confirms being the sole contributor of this work and has approved it for publication.
